# Bayesian interpretation of p values in clinical trials

**DOI:** 10.1136/bmjebm-2020-111603

**Published:** 2021-09-23

**Authors:** John Ferguson

**Affiliations:** Clinical Research Facility, National University of Ireland Galway, Galway, Ireland

**Keywords:** clinical decision-making, methods

## Abstract

Commonly accepted statistical advice dictates that large-sample size and highly powered clinical trials generate more reliable evidence than trials with smaller sample sizes. This advice is generally sound: treatment effect estimates from larger trials tend to be more accurate, as witnessed by tighter confidence intervals in addition to reduced publication biases. Consider then two clinical trials testing the same treatment which result in the same p values, the trials being identical apart from differences in sample size. Assuming statistical significance, one might at first suspect that the larger trial offers stronger evidence that the treatment in question is truly effective. Yet, often precisely the opposite will be true. Here, we illustrate and explain this somewhat counterintuitive result and suggest some ramifications regarding interpretation and analysis of clinical trial results.

## Introduction

Imagine a scenario where a phase III clinical trial comparing drug X to a placebo control is terminated early due to insufficient patient recruitment. Treatment allocation is subsequently unblinded to the trial statistician and statistical analysis ensues. Unfortunately, the cat is now out of the bag: the protocol has been violated and there are concerns that conclusions may be comprised due to insufficient statistical power. Due to these concerns, it is decided to retrospectively downgrade the trial to exploratory phase II status.

However, when the analysis results are uncovered, there is a shock: a significant p value, just beneath 0.05. The study primary investigator is frustrated; a larger sample size (as per protocol) with the same p value would have constituted evidence of drug X’s effectiveness that might be acceptable to a regulator, rather than the more exploratory conclusions that a phase II trial allows.

While the above story is just an anecdote and would be an unusual circumstance for most clinical trial teams, it might capture to some degree a misunderstanding within the clinical community. In fact, there is no general statistical justification for the thinking that an equal p value represents stronger evidence that the treatment is effective in the larger trial compared with the smaller trial. In this article, we explain this statement from an intuitive, non-mathematical viewpoint.

## P values and posterior probabilities

Informally speaking, a p value reflects the (long-run) probability of observing the effect seen in the sample data, or a more extreme effect, if in fact there is no treatment effect. A Bayesian calculation instead estimates the probability that there *is* a treatment effect after seeing the data from the trial. This second quantity is called a ‘posterior probability’. Posterior probabilities have a more intuitive interpretation than p values as reflected by students sometimes misinterpreting p values as posterior probabilities when learning introductory statistics.^1^ In addition, we will explain later that posterior probabilities can reflect a more complete summary of the evidence for or against the null hypothesis of no treatment effect than p values, provided they are calculated using an appropriate prior distribution.

## Prior distributions for the treatment effect parameter

**Figure 1:**
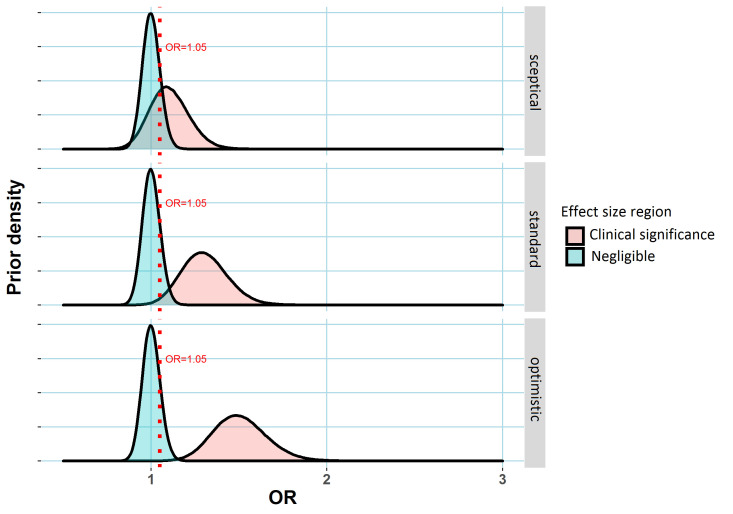
Three possible prior distributions for a treatment effect (measured via an OR relative to a placebo control) in a clinical trial. In each case, the region shaded blued represents values of the treatment effect that might be regarded as clinically unimportant. The dashed red line represents an OR of 1.05, the assumed minimum clinically meaningful treatment effect. The alternative region (shaded red) represents plausible treatment effect sizes under the alternative scenario the treatment has an important effect. Three hypothetical alternative effect size distributions are specified corresponding to differing prior opinion regarding the drug’s putative effectiveness, labelled ‘sceptical’, ‘standard’ and ‘optimistic’, corresponding to expected ORs of 1.1, 1.3 and 1.5 are shown here. to complete prior specification, the analyst needs to specify the prior probability of a clinically important treatment effect. This probability is assumed to be 20% here (implying the prior probability that the treatment is ineffectual is 80%). Created by the author. OR, odds ratio.

The prior distribution summarises the investigator’s belief about the true value of the treatment effect before collecting data. Figure 1 uses the ‘lump and smear’ approach from^2^ to specify three possible priors, each representing differing beliefs regarding the extent of the treatment’s efficacy. The approach involves subjective specification for the probability that the treatment is effective before seeing any data (the prior probability of a treatment effect), a statistical distribution of possible values for the size of treatment effect if the treatment were effective, and a distribution of effect sizes that would be considered clinically unimportant or ineffectual (see the figure 1 legend for more details). In doing this, one has to consider how to measure treatment effects. For numeric outcomes (for instance, blood pressure), often treatment effects could be more simply measured as differences in the mean outcomes in the two arms. Here, we consider binary-valued outcomes (such as 1-year mortality or progression of disease) and will measure treatment effects as odds ratios (ORs), which provided the outcome is rare can be interpreted as a ratio of event rates in the placebo and treatment arms, ORs close to 1 indicating negligible treatment effects. The necessity of choosing such a prior distribution is an advantage of the Bayesian framework since that allows incorporation of scientific information external to the data into the analysis. However, it may also be viewed as a disadvantage as often an appropriate prior will be difficult to specify. For instance, perhaps different experts might have differing opinions on a drug’s effectiveness, in which case a particular choice of a prior corresponding to a single individual’s belief adds a degree of subjectivity to the ensuing analysis.

Having specified a prior distribution, a Bayesian asks again ‘what is the probability that there is a treatment effect, given the dataset that has resulted?’. With prior distributions specified as in figure 1, this is the data-updated probability that the true OR lies in the region of treatment effect values shaded red. Statistically, this probability is known as the posterior probability of a treatment effect.

## Bayesian interpretation of evidence at a fixed p value for different sample sizes

**Figure 2:**
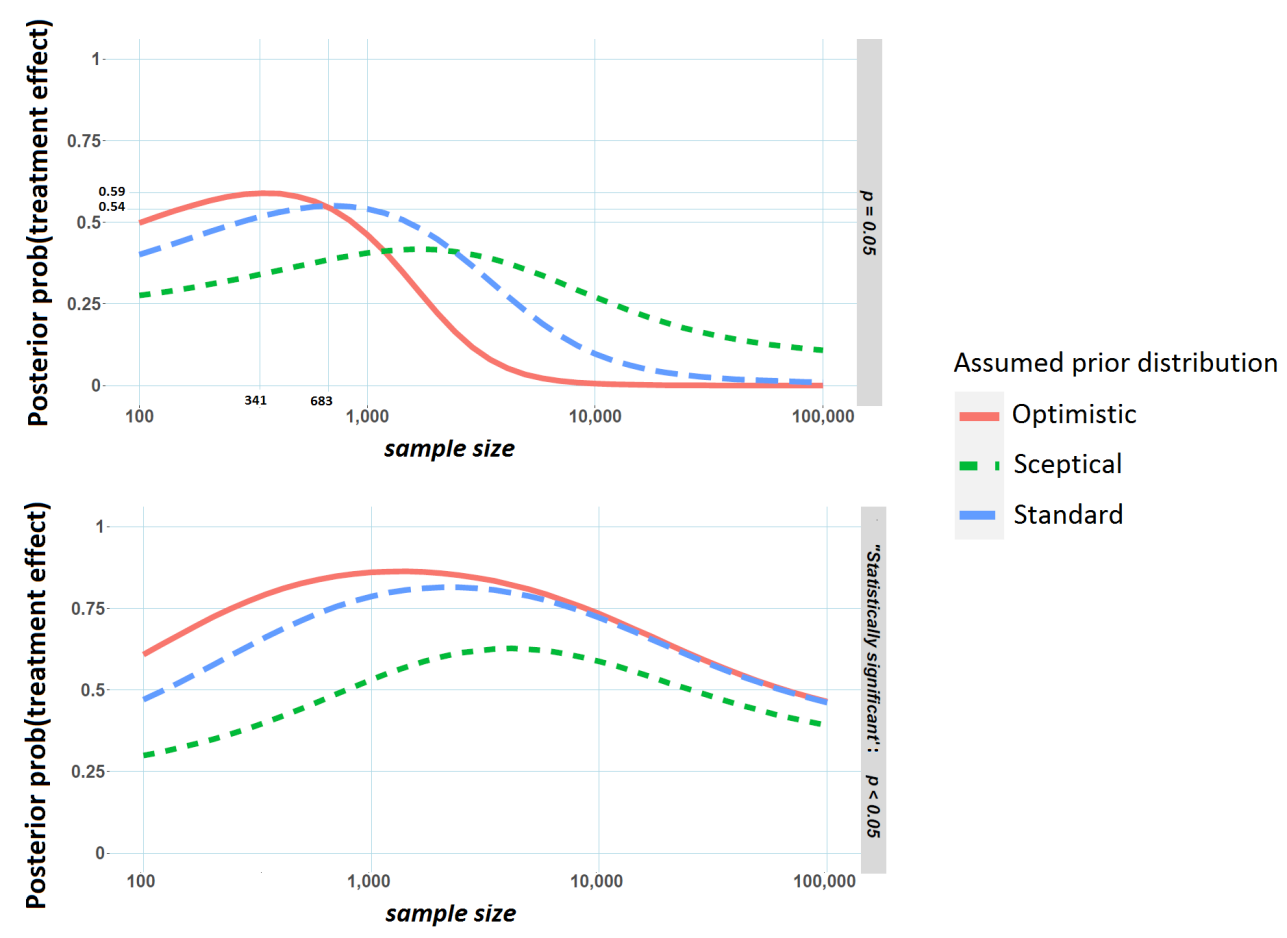
Posterior probability of a treatment effect versus sample size (per arm) in the clinical trial. Results are from simulated data where the event rate in the placebo arm is 10%. The upper panel represents the probability of a treatment conditional on a p value of 0.05. The lower panel shows the probability of a treatment effect conditional on a significant p value at the 5% level. Created by the author.

The upper panel of figure 2 displays this posterior probability at different sample sizes for the three priors (‘sceptical’, ‘standard’ and ‘optimistic’) in figure 1, assuming an event rate in the placebo arm of 10% and a fixed p value of 0.05. As an example, suppose the trial statistician (referred to in the introduction) has derived a sample size of 683 per arm, sufficient to detect a difference in event rates of 5% (10% vs 15%) between drug X and the placebo with 80% power. Suppose also that the optimistic prior in figure 1 represents the investigator's belief about the drug’s effectiveness (note that this ‘optimistic’ prior suggests an OR of about 1.5, which is consistent with the power calculation, if there is an effect). If the trial is stopped early after only recruiting 341 patients per arm, and the p value for testing the difference in the proportion of event rates between the two arms is 0.05, the posterior probability of a treatment effect can be shown to be 59%. If on the other hand, the trial was not stopped early, that is the total 683 patients per arm are recruited, the posterior probability of a treatment effect is only 54% if a p value of 0.05 is observed.

In particular, for the standard and optimistic priors, the probability of a treatment effect tends to decrease as the sample size gets larger. An intuitive explanation for this pattern is that under the optimistic prior we would expect to see relatively large observed effect sizes (and low p values) if there truly is a treatment effect. Increasing sample size and not finding this expected result (that is a p value far beneath 0.05) may dampen this initial optimism. More technically, the posterior probability depends not only on how likely a p value of 0.05 would be assuming no treatment effect but also how likely a p value of 0.05 is assuming there is a treatment effect. As the sample size gets larger and larger, the first probability remains unchanged (p values always have a flat distribution when there is no treatment effect, regardless of sample size) but the second probability will get quite small with values much smaller than 0.05 becoming increasingly likely so that posterior probability of a treatment effect given a p value of 0.05 actually gets smaller as the sample size increases.

## Correcting overestimation in smaller trials

**Figure 3:**
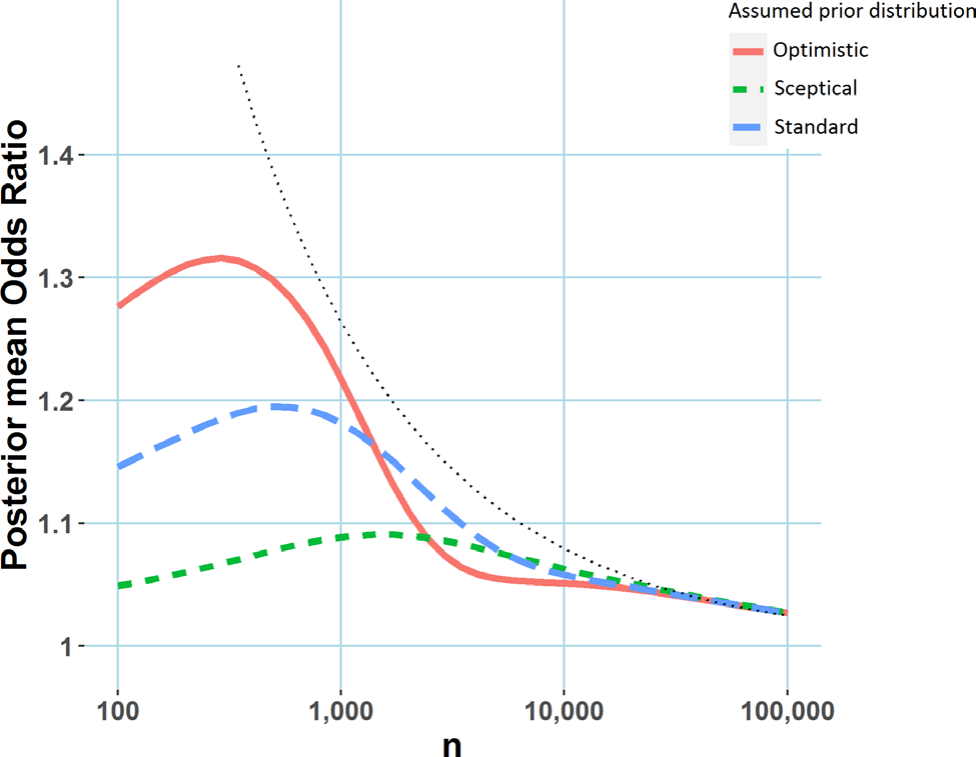
Posterior mean OR versus sample size (per arm), conditional on a p value of 0.05, for the priors illustrated in figure 1. Results are from simulated data where the event rate in the placebo arm is 10%. The dotted black line represents the observed OR necessary to achieve a p value of 0.05 at various sample sizes. Created by the author.

An observed effect size corresponding to a significant p value may well be reflective of both a large true OR and random chance, because a p value is more likely to be small if the associated data are the result of both a large true OR and random chance acting in the direction of exaggerating that OR. As a result, effect size estimates corresponding to significant p values tend to be upwardly biased. Since random chance plays a larger role in smaller trials, the bias will typically be larger for smaller trials.^3^ However, a Bayesian analysis using an appropriate prior can mitigate this bias to a degree. From a Bayesian point of view, the posterior distribution (a probability distribution like those shown in figure 1, but which combines the chosen prior with the data) incorporates all information about the treatment effect after seeing the data,^4^ and as a result the average value for this distribution (ie, the posterior mean) is an unbiased estimate of the treatment effect.^5^ ^6^ Posterior mean ORs for differing sample sizes are illustrated in figure 3, for the priors (‘sceptical’, ‘standard’ and ‘optimistic’) illustrated in figure 1. Note the posterior mean ‘shrinks’ the observed OR toward 0, which can be regarded as a Bayesian bias correction. Interestingly, for a fixed p value of 0.05, the posterior mean OR is sometimes larger for smaller sample sizes, indicating that even after correcting for the significance bias (sometimes known as the winner’s curse or publication bias^7^), we would expect the true effect corresponding to a p value of 0.05 to be larger for smaller trials.

## The replication crisis and the distrust of small trials

For better or for worse, p values are often compared with significance thresholds, and most often a 5% threshold, to make decisions. How likely is an important effect when we observe a significant p value (p≤0.05) and ‘reject the null’? This second posterior probability is closely related to the percentage of significant results that would replicate in high-powered follow-up studies and thus the reproducibility of science.^8^ In the examples demonstrated in the lower pane of figure 2, this probability initially increases as the sample size increases. This is a well-known result,^9^ and stands to reason. As trial sample size increases, a significant p value becomes more and more likely when there is an important treatment effect (ie, statistical power will increase), but this increase is far less pronounced for negligible treatment effects. The posterior probability of an important treatment effect having observed a significant p value depends on the ratio of these two probabilities, and so will initially increase with sample size. However, studies with extremely large sample sizes are likely to yield significant p values even for small, clinically unimportant effects. Reversing this logic, attainment of statistical significance in large studies is not necessarily indicative of a clinically important treatment effect; a phenomenon that explains the dip in posterior probability for large sample sizes.

## What is the correct prior to use?

As mentioned earlier, there is often justifiable uncertainty regarding the choice of an appropriate prior. This is important to note as for a fixed p value, the relationships between sample size and Bayesian evidence for a treatment effect do depend somewhat on this background knowledge. For instance, the pattern in figure 2 is quite different for the sceptical prior, compared with the standard and optimistic priors. With no acceptable consensus view or no historical data that could be used to construct a prior, it is difficult to link a p value to any measure of Bayesian evidence. However, if scientific or subjective knowledge regarding the effect of interest is unavailable, another possibility is to use a prior that roughly corresponds to the distribution of true effect sizes in similar experiments, or indeed in a field of interest. This is the approach applied roughly here where 20% prior probability was attributed to larger interesting effect sizes in the alternative region, shaded red in figure 1.^10^ If this ‘field-related’ prior was somehow known exactly for a particular area of science, and used by practitioners in their analyses, the resulting inferences would go a long way toward solving the replication crisis, highlighted by Ioannidis.^11^ For instance, out of the studies that concluded a 95% probability of an important effect when using such a prior, only 5% of these would be false positive, again using the result that Bayes estimates, calibrated with an appropriate prior, are immune to selection bias.^5^ In practice though, many if not most statistically insignificant results are supressed and good data to estimate such a prior is hard to obtain. A related and important point is that smaller trials tend to be more exploratory and perhaps deserve a more sceptical prior (in the sense of figure 2) than larger confirmatory phase III trials. In other words, we might distrust the veracity of a p value of 0.05 in a small phase II trial primarily because we were sceptical of whether an effect existed in the first place, rather than because the sample size was low.

## Implications for analysis and interpretation

Despite justifiable concerns regarding the way they are employed,^12^ p values are likely to remain popular in clinical trials where regulatory bodies are suspicious of analyses that do not control long-run statistical properties such as type I error and statistical power. Perhaps Bayesian analyses, similar to those we present here, should be more encouraged by regulators, even if p value thresholds are used in approval decisions. In this endeavour, regulators could possibly help in specifying prior distributions, so as to assuage the concern that an investigator-chosen prior distribution might bias results. Such additional reporting has the potential to increase the interpretability of trial results since for the reasons explained above Bayesian methods can give a broader overview of the evidence for or against a treatment effect.

## In conclusion

We now recall the dilemma of the frustrated investigator mentioned in the introduction. Their misconception was that a p value of just beneath 0.05 was somehow unreliable given that ongoing trial was stopped early due to insufficient patient recruitment. However, we have argued here that without formally eliciting a prior for the OR, and assuming that early stopping of the trial should in no way dampen the investigator's belief regarding the drug’s effectiveness, there is no good statistical reason why conclusions should be different than if the same p value was observed in a larger trial. Of course, when planning the trial a larger sample size would have be better. Larger sample sizes result in improved power of finding an important treatment effect when it’s there, as well as tighter CIs, and reduced publication biases when an effect is significant; but pre-experimental planning and statistical inference given a borderline significant p value are quite different things.

The results explained here regarding the relationships between p values, Bayesian evidence for a treatment effect and sample size are well known.^13^ In a way, they are unsurprising, as a p value (which examines the compatibility of the data with the null hypothesis of no-treatment effect) on its own only gives partial information regarding the potential truth of the alternative hypothesis that the treatment is effective. We alluded to this earlier when we observed that while such a p value considers the distribution of the test statistic under the null distribution of no treatment effect, it ignores the distribution under the alternative hypothesis of an effective treatment, and the consideration of both are necessary to thoroughly summarise the evidence in the data regarding whether the treatment is effective. In contrast, Bayesian procedures incorporate both these distributions implicitly and consequently can offer a more complete view of the evidence for or against a treatment effect, incorporating pre-experimental knowledge with efficient utilisation of observed data. Incorporating such thinking into clinical trial analyses where possible could help avoid possible misconceptions resulting from relying on p values alone.
